# Changes in Two Point Discrimination and the law of mobility in Diabetes Mellitus patients

**DOI:** 10.1186/1749-7221-3-3

**Published:** 2008-01-29

**Authors:** R Periyasamy, M Manivannan, Vengesana Balakrish Raja Narayanamurthy

**Affiliations:** 1 Biomedical Engineering Group, Department of Applied Mechanics, Indian Institute of Technology Madras, Chennai, 600036, India; 2 Diabetic Foot Clinic, Sundaram Medical Foundation, Chennai, 600040, India

## Abstract

**Background:**

Diabetic neuropathy is a family of nerve disorders with progressive loss of nerve function in 15% of diabetes mellitus (DM) subjects. Two-point discrimination (TPD) is one method of quantitatively testing for loss of nerve function. The law of mobility for TPD is known for normal subjects in earlier studies but has not been studied for diabetic subjects. This is a pilot study to evaluate and plot the law of mobility for TPD among DM subjects.

**Methods:**

The Semmes Weinstein monofilament (SWMF) was used to measure the loss of protective sensation. An Aesthesiometer was used to find the TPD of several areas in upper and lower extremities for normal and diabetic subjects. All the subjects were screened for peripheral artery occlusive disease with ankle brachial pressure index (0.9 or above).

**Results:**

TPD of normal and diabetic subjects for different areas of hands and legs from proximal to distal is evaluated for 18 subjects. TPD values decrease from proximal to distal areas. Vierodt's law of mobility for TPD holds good for normal subjects in the hand and foot areas. The law of mobility for TPD in DM subjects holds well in the hand but doesn't hold well in foot areas with or without sensation.

**Conclusion:**

TPD is a quantitative and direct measure of sensory loss. The TPD value of diabetic subjects reveals that the law of mobility do not hold well for Diabetic subjects in foot areas. The significance of this result is that the TPD of the diabetic subjects could provide direct, cost effective and quantitative measure of neuropathy.

## Background

Foot problems are the most common reason for hospitalization of diabetes mellitus (DM) patients. Neuropathy and impaired blood supply are the chief causes for foot ulceration in DM patients. In the presence of neuropathy, the primary factor for ulcer formation in diabetes is the loss of protective sensation [1,2]. Evaluation of sensibility on the feet of diabetic patients is important in order to properly identify the group with neuropathy and to establish prevention of ulceration for those at risk. Various modalities of sensation like temperature, vibration, point localization and two point discrimination (TPD) have been used to measure sensory loss. Semmes-Weinstein monofilament testing (10 gm) divides the huge population of DM subjects into subjects who are at risk but does not precisely determine the degree of progressive loss of sensation, or suggest degree of nerve compression and axonal loss. Specialist clinics may quantify neuropathy with biothesiometry, plantar foot pressure measurement, and assess lower extremity vascular status with handheld Doppler systems and ankle-brachial blood pressure indices (ABPI).

Though monofilament testing is one of the primary screening methods for measuring cutaneous sensibility clinically, the sensibility can be only qualitatively assessed as normal touch, diminished light touch, diminished protective sensation, and loss of protective sensation [3] depending upon the size of the filament and force exerted to buckle the mono-filament. The measurement of cutaneous sensation to differentiate one-point from two-point static touch stimuli may allow identification of ulceration earlier in the clinical course of diabetic neuropathy [4]. Static and moving TPD have been used as tools to measure sensory loss in DM patients. Although the method is subjective, as the patient must report whether or not the pressure is felt, it is more reliable than the previously available methods and it is a quantitative measure of the sensory loss. Both the vascular dysfunction and the neuropathy result in increased TPD in foot areas. Therefore, increase in TPD does not necessarily indicate either neuropathy or vascular dysfunction [5].

It is well known that TPD obeys the law of mobility in normal subjects [6], however the applicability of the law to diabetic subjects is not known. This paper presents the first systematic study of the law of mobility to assess the sensibility of diabetic subjects, comparing the law of mobility of TPD in the upper and lower extremities of DM patients. We observe that the law of mobility does not hold well in DM patients.

### Law of Mobility

Research on cutaneous sensibility was undertaken in the nineteenth century by Vierodt [6] and Weber [7]. Weber introduced the point localization test and the accompanying measures, two-point discrimination (TPD) and localization error, as measures of cutaneous sensibility. Density of mechanoreceptors in an area determines the TPD. A dense population leads to finer TPD and the receptors have smaller receptive fields. Mapping of the whole body revealed large differences in the sensibility between different parts of the body. Vierodt generalized this observation into the '*law of mobility*', which states that the *TPD improves with the mobility of the body part*. TPD correlates with the Degree of Freedom (DOF) of the body part. It is to be noted that no exception to this law has been found yet. After the work of Weber and Vierodt, little attention was given to this field until the 1950s [8] and 1960s [9]. Weinstein observed significant effects of body locus. Lowest TPD was found for the fingertips (2.5 mm). TPD for the trunk was approximately 40 mm. Sensitivity decreased from distal to proximal regions, and thresholds correlated with the relative size of cortical areas subserving a body part. Another important observation was that good TPD did not necessarily mean good sensitivity to pressure, that is, a low detection threshold.

### Static and dynamic Two-Point Discrimination

The minimum distance between two stimulus points on the skin, which are perceived as distinct points, is defined as TPD. Among the two types of TPD, static and dynamic TPD, static two-point discrimination (STPD) is commonly used in emergency departments to determine digital nerve integrity [10]. Static TPD is the current recommended method for physicians evaluating degree of sensory loss in diabetic patients. Dynamic TPD (DTPD) is usually measured with a Disk-criminator [10], moving the prongs along the surface of the center. Moving TPD values were smaller in magnitude than stationary TPD values in all anatomical areas tested. Dynamic TPD is not routinely used in clinical practices. In this paper we consider only the static two-point discrimination for the law of mobility.

### Methods to measure TPD

Calipers or an opened paper clip with two parallel ends are used for finding STPD [11]. An aesthesiometer is a modified form of vernier caliper useful for determining the TPD of touch, by moving the prongs into contact with the portion of the body part and then pressing until the patient feels a sensation. A disk-criminator, consisting two rotating plastic disks that are joined together, is useful for testing DTPD [12]. A set of small grating surfaces recently introduced for cutaneous spatial resolution measurement. The gratings are placed on the skin and subjects are required to identify the orientation of grooves and bars. The finest grating whose orientation is discriminated reliably provides an estimate of the spatial resolution limit in the tested area [13]. In the 1990s, Dellon proposed the Pressure-Specified Sensory Device (PSSD) that could gather information about static and moving touch in a continuous form by using a computer [12]. PSSD is a quantitative sensory device, which consists of one or two blunt probes and sensitive transducers to measure and record the perception thresholds of pressure on the surface of the body in gms/sqmm. PSSD is not routinely used in clinical practices. In this paper we used an aesthesiometer to measure the TPD.

## Methods

We measured the loss of protective sensation and TPD in forearm, palm, fingers, lower leg, and foot areas. While the loss of protective sensation was measured using 10 gm SWMF [3], TPD was assessed using an aesthesiometer. We tested the pressure exerted by two prongs of the aesthesiometer using a weighing balance. A total of 50 gm was exerted on usual pressure.

**Figure 1:**
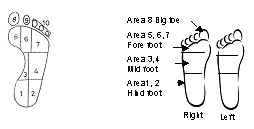
a Standard Division of foot area. b Division of foot area for our study.

Although in literature the foot is divided into ten standard significant areas as shown in figure 1(a) as per method indicated in [1,14], for our analysis, we divided the foot into four areas as shown in figure 1(b). Hind foot combines areas 1 & 2, mid foot combines areas 3 & 4, fore foot combines areas 5, 6 & 7, and the big toe is area 8.

We evaluated sensibility of the feet of 18 subjects. Sensibility values for the normal subjects were collected from the literature and four normal subjects were also included in our study. The fourteen other subjects were DM patients.

The test was carried out with the patient in a comfortable reclining position with eyes closed. SWMF (10 gm) was used on the different areas of the foot to find the sensation. An aesthesiometer was used to find the TPD. The two prong tips of aesthesiometer were made to touch the body part at the same instant. The subject orally stated whether he/she perceives the touch as a single point or as two separate points. Occasionally, without the subject's knowledge, the subject was touched with only one prong. This prevented the subject from knowing whether or not a two-point stimulus was always delivered. When the subjects consistently perceive one point rather than two points, the TPD is reached and this was recorded in the datasheet.

If the subject had callosity in any of the foot area, the TPD measurement was taken in the adjacent area to the callosity but within the same area of the foot. The subject's age, duration of the DM, medication were noted but not considered for our analysis. Other than the foot area, we also tested TPD in lower legs and similarly three areas (forearm, palm, fingers) in the upper extremities.

The study period was from Jan'07 to Mar'07. A total of 18 subjects were tested and the details of diabetic subjects are given below in the table 1. All the subjects were screened for peripheral artery occlusive disease (PAOD) with the ankle-brachial-blood pressure index (0.9 or above).

**Table 1:** Details of Diabetic Subjects

Diabetic subjects		Age of Diabetic subjects	Number of subjectswith callosity	Duration of diabetes mellitus	
Male	Female			Male	Female
			
5	9	50 – 70	3	5–20	5 – 20

## Results

Subjects are classified as *with sensation *and *without sensation *based on their response to SWMF i.e. able to feel 10 gm monofilament. Of the 14 DM subjects, 5 subjects had sensation and 9 subjects did not have sensation. The values for TPD used for plotting the graphs represent the mean value of TPD.

### Normal and diabetic TPD for legs

**Figure 2:**
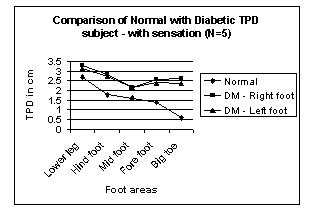
Comparison of Normal with Diabetic TPD – with sensation in Leg areas. N → number of subjects.

**Figure 3:**
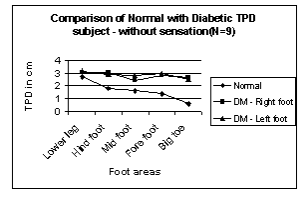
Comparison of Normal with Diabetic TPD – without sensation in Leg areas. N → number of subjects.

Figures 2 and 3 show TPD of normal and diabetic subjects for different areas of leg and foot from proximal to distal.

In normal subjects, the TPD value from the leg to big toe decreases from 3 cm to 0.5 cm. But in diabetic subjects the TPD value decreases from 3 cm to 2.5 cm. It suggests that Vierodt's law of mobility holds well in the leg areas for the normal subjects. The lower leg has less mobility than the foot. The TPD of the lower leg is more than that of the hind foot. The foot area i.e. hind foot, mid foot and forefoot have similar mobility monotonically increasing from proximal to distal and their TPD is almost the same. However, the mobility of the big toe is more and its TPD is significantly smaller than that of previous areas. This same trend is observed in both left and right legs. Therefore, the law of mobility doesn't hold for the DM subjects with sensation as shown in figure 2. Figure 3 shows TPD values for DM subject without sensation and the results are similar to those with sensation.

### Normal and diabetic TPD for hands

**Figure 4:**
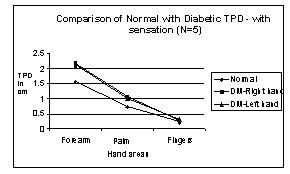
Comparison of Normal with Diabetic TPD – with sensation in Hand areas. N → number of subjects.

**Figure 5:**
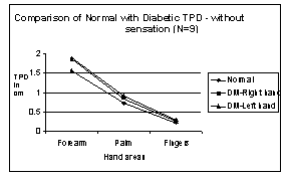
Comparison of Normal with Diabetic TPD – without sensation in Hand areas. N → number of subjects.

We tested the law of mobility in the upper extremities. Figures 4 and 5 show TPD of normal and diabetic subjects for different areas of the hand from proximal to distal. It is observed from the graph that TPD values decrease from proximal to distal areas and that the law of mobility holds for both normal and DM subjects with and without sensation in both right hand and left hand.

## Discussion

**Figure 6:**
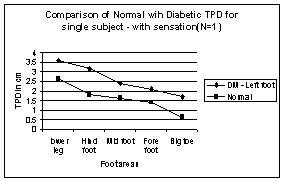
Comparison of Normal with Diabetic TPD for single subject – with sensation in Left Leg areas. N → number of subject.

As expected, the TPD of DM subjects is always higher than that of normal subjects, at least in some areas of the foot. Though the TPD values expressed here in all the above graphs are mean of subjects with sensation and without sensation, the law of mobility is not obeyed even if we evaluate individual DM subjects. This is true for both right and left legs for all the subjects, except for one subject who had sensation, whose left leg obeys the law of mobility as shown in figure 6.

This observation in the foot areas with and without sensation is not true in the hands of DM subjects; the law of mobility holds well in the hand of the DM subjects. As Rendell [15] have stressed, the maldistribution between nutritional and thermoregulatory skin blood flow is observed in the toes but not the fingers of diabetic patients. This could be directly related to the development of ulcers in the feet but not in the hands.

Urbancic [5] describes the role of micro vascular dysfunction in the development of diabetic foot ulceration and the differences between the left and right, and the lower and upper extremities. In another study, Joseph [16] describes the decline of tactile acuity in aging due to blood-supply and other factors. Such micro vascular dysfunction could have resulted the differences in the TPD between left and right, and the lower and upper extremities of the DM subjects. Measure of TPD and the law of mobility in DM subjects could then easily reveal such micro vascular dysfunction and its role in neuropathy. TPD measurements therefore could give quantitative measure of axonal loss in compression neuropathy.

**Figure 7:**
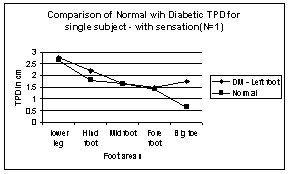
Comparison of Normal with Diabetic TPD for single subject – with sensation in Left Leg areas. N → number of subject.

**Figure 8:**
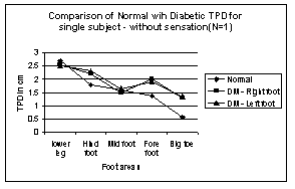
Comparison of Normal with Diabetic TPD for single subject – without sensation in Leg areas. N → number of subject.

One of the subjects had the TPD values merely increased (from normal value) uniformly over all the areas in the left leg, still satisfying the law of mobility, typical sign of micro vascular dysfunction is shown in figure 7. Another subject had the same TPD values as normal subjects except in one-foot area, typical sign of neuropathic condition is shown in figure 8. Although all the subjects were screened for peripheral artery occlusive disease (PAOD) with the ankle-brachial-pressure index (0.9 or above), the test does not completely eliminate the subjects with micro vascular dysfunction from the experiment. Most of the DM subjects have neuro-ischaemia problems, simultaneously with neuropathy and micro vascular dysfunction.

In this paper we have presented the law of mobility only for TPD. It is well-established fact that point localization has direct correlation with TPD, though not the same [17]. Similarly SWMF has direct correlation with the point localization, as the later measures the area localisation error and the SWMF measure pressure threshold in terms of weight. It is possible that the law of mobility could be used with point localisation and SWMF as well. The other sensory measures such as vibration detection threshold (VDT), cold detection threshold (CDT), warm detection threshold (WDT), and heat pain onset threshold (HPO) could be studied for the law of mobility, specifically in DM subjects.

## Conclusion

Although TPD measurement is subjective, it is a well-established test to measure sensory loss in DM subjects. This paper presents the first systematic study of the law of mobility to assess the sensibility of diabetic subjects, comparing the law of mobility of TPD in upper and lower extremities of DM patients. We observe that the law of mobility does not hold well in DM patients.

The TPD data for diabetic patients reveals that the law of mobility for diabetic patients does not hold in the foot areas. The significance of this result is that the TPD of the diabetic patients could provide direct and quantitative measure of micro vascular dysfunction and its effect on neuropathy. Though TPD has been accepted widely as a measure of sensory loss, we did not find any particular difference in the applicability of the law for DM subjects with and without sensation of 10 gm monofilaments.

We screened the subjects for peripheral artery occlusive disease (PAOD) with the ankle-brachial-pressure index (0.9 or above), but this test does not completely eliminate the subjects with micro vascular dysfunction. However more accurate techniques like laser doppler flow, transcutaneous oxygen saturation, skin temperature or any combination of these techniques could better evaluate the use of law of mobility for microvascular dysfunction.

The law of mobility shows the degree of axonal loss. The law of mobility for TPD of diabetic subjects may provide a simple, easy, cost effective clinical tool to evaluate compression neuropathy in patients with or without neuropathy as shown by SWMF and progressive axonal loss due to microvascular dysfunction subsequent to tarsal tunnel compression. If the sensory loss is due to microvascular dysfunction because of compression neuropathy at the tarsal tunnel, changes of law of mobility towards normal will indicate success of decompression of the nerve at the site of compression.

## Authors' contributions

All the authors had full access to all data in the study. VBN carried out the clinical studies and participated in drafting the manuscript. Both MM and PR conceived of the study and participated in its design and coordination. All authors read and approved the final manuscript.
